# Advances in High-Field Magnetic Resonance Spectroscopy in Alzheimer’s Disease

**DOI:** 10.2174/1567205011666140302200312

**Published:** 2014-05

**Authors:** Ningnannan Zhang, Xiaowei Song, Robert Bartha, Steven Beyea, Ryan D’Arcy, Yunting Zhang, Kenneth Rockwood

**Affiliations:** 1 National Research Council Canada, Institute for Biodiagnostics – Atlantic, Halifax, Nova Scotia, Canada;; 2 Department of Radiology, General Hospital of Tianjin Medical University, Tianjin, China;; 3 Division of Geriatric Medicine, Department of Medicine, Dalhousie University, Halifax, Nova Scotia, Canada;; 4 Neuroimaging Research Laboratory, Biomedical Translational Imaging Centre, Halifax, Nova Scotia, Canada;; 5 Centre for Functional and Metabolic Mapping, Robarts Research Institute, University of Western Ontario, London, Ontario, Canada;; 6 Department of Medical Biophysics, University of Western Ontario, London, Ontario, Canada;; 7 Department of Physics, Dalhousie University, Halifax, Nova Scotia, Canada;; 8 Department of Applied Science, Simon Fraser University, Surrey, British Columbia, Canada;; 9 Surrey Memorial Hospital, Fraser Health Foundation Innovation, Surrey, British Columbia, Canada;; 10 Centre for Health Care of the Elderly, Queen Elizabeth II Health Sciences Centre, Halifax, Canada

**Keywords:** Alzheimer’s disease, brain, high-field, metabolite, mild cognitive impairment, neurochemical, proton magnetic resonance spectroscopy.

## Abstract

Alzheimer’s disease (AD) affects several important molecules in brain metabolism. The resulting neurochemical changes can be quantified non-invasively in localized brain regions using in vivo single-voxel proton magnetic resonance spectroscopy (SV 1H MRS). Although the often heralded diagnostic potential of MRS in AD largely remains unfulfilled, more recent use of high magnetic fields has led to significantly improved signal-to-noise ratios and spectral resolutions, thereby allowing clinical applications with increased measurement reliability. The present article provides a comprehensive review of SV 1H MRS studies on AD at high magnetic fields (3.0 Tesla and above). This review suggests that patterned regional differences and longitudinal alterations in several neurometabolites are associated with clinically established AD. Changes in multiple metabolites are identifiable even at early stages of AD development. By combining information of neurochemicals in different brain regions revealing either pathological or compensatory changes, high field MRS can be evaluated in AD diagnosis and in the detection of treatment effects. To achieve this, standardization of data acquisition and analytical approaches is needed.

## 1. INTRODUCTION

Alzheimer’s disease (AD), the most common cause of dementia in late life [1], is characterized by an insidious onset and progressive neurodegeneration [2]. The hallmark accumulation of extracellular amyloid plaques and intracellular neurofibrillary tangles typically start in the entorhinal cortex and the medial temporal lobe (MTL) and extend gradually to the entire neocortex [2]. The clinical diagnosis of AD now emphasizes coupling comprehensive clinical examinations with biomarkers, including brain imaging [3, 4]. To date, AD treatments are symptomatic and administered only when the disease is established. There is a strong belief that real progress will require early administration of disease modifying interventions – given perhaps even before clinical symptoms [5-7]. Such an ambitious undertaking cannot be realized without some way of detecting neuropathological changes *in vivo*. 

*In vivo* magnetic resonance spectroscopy is an evolving non-invasive neuroimaging method that can be used to detect changes in neurometabolites in the living brain, thereby allowing neuropathological deficits to be linked to cognitive decline [8-10]. The single voxel proton magnetic resonance spectroscopy (SV^ 1^H MRS) has been by far the most frequently used technique in studying AD-associated changes of neurometabolites. Existing reviews mostly have reported findings using low-moderate magnetic fields (*e.g.*, 1.5 Tesla) published until 2007 [11-14], whereas recent studies chiefly employ 3.0 T and above; these contributions have been less well summarized [15-17]. Indeed high-field MR systems are becoming the benchmark in research and clinical settings, as they can provide increased spectral signal-to-noise ratio (SNR) and chemical shift dispersion [18, 19]. Even so, the high-field MRS findings have yet to be appraised. 

This article reviews high-field SV ^1^H MRS in AD. Our specific objectives were to update the evidence regarding (1) AD-characteristic changes of several important brain metabolites, (2) the spatial (brain regions) and phase (disease stage) dependent changes of multiple metabolites, and (3) important metabolite changes in response to treatment. We conclude by describing the potential clinical value and direction of MRS in the study of AD.

## 2. METHODS 

### 2.1. Search Terms 

We searched the MEDLINE database using terms that combined the following three sets of phrases. Set-1: “Alzheimer’s disease”, “mild cognitive impairment”, or “dementia”; set-2: “magnetic resonance spectroscopy”, “proton MRS”, “nuclear MRS”, or “MRS imaging”; set-3: “brain”, “cortex”, “cortical”, “grey matter”, or “white matter”. Examples of the search terms included “Alzheimer’s disease AND magnetic resonance spectroscopy AND brain”, “mild cognitive impairment AND magnetic resonance spectroscopy AND brain”, and “Alzheimer’s disease AND proton MRS AND cortex”, and so on. Field strength was not designated as a search term; thus studies conducted at all field-strengths were made available for further filtering.

### 2.2. Inclusion and Exclusion Criteria 

Initially, the search yielded 707 individual articles, published between 1987 (time of the first MRS in dementia publication in the literature) and January 31, 2014. All retrieved articles were filtered by reading the abstracts, with selections based on the following inclusion and exclusion criteria. Inclusion: (1) Peer-reviewed journal articles that studied and/or reviewed MRS in AD, dementia, MCI, and normal aging; (2) Studies that applied a MRS method, including *in vivo* SV ^1^H MRS, magnetic resonance spectroscopy imaging (MRSI, to allow simultaneous acquisition from multiple regions, although signal contamination can be a concern), phosphorous spectroscopy (^31^P MRS, to detect changes in phospholipid metabolism, although with lower sensitivity) and carbon spectroscopy (^13^C MRS, to detect changes in glucose to glutamate conversion). Exclusion: (1) studies that did not involve any MRS method; (2) articles that did not involve *in-vivo* investigations on humans*; *and (3) articles published in languages other than English. This processing resulted in a subset of 245 articles, including 190 original research reports. Most of these studies (including all at high-fields) used non-invasive SV^ 1^H MRS. The 27 high field studies (at 3.0 Tesla and above) were reviewed in greater detail. 

## 3. TECHNICAL REMARKS

### 3.1. Basics* of In Vivo* SV ^1^H MRS

Single voxel ^1^H MRS is designed to assess metabolic information from specific brain regions, based on the magnetic resonance properties of the hydrogen proton [8-10]. This is the MRS method most widely used in *in vivo* AD studies; only a few investigations have used ^31^P, ^13^C MRS, or MRSI. A brief overview of key aspects of SV ^1^H MRS is provided next, to familiarize readers with this technique.

#### 3.1.1. Chemical Shift 

When placed in a magnetic field, hydrogen nuclei resonate at a characteristic frequency. When excited in the magnetic field, each hydrogen nucleus within a metabolite experiences a very small shift in resonance frequency (chemical shift; expressed in parts per million, ppm) due to multiple factors in the chemical environment (*e.g.*, atom negativity, electron density, magnetic field strength) [20]; this very small shift forms the basis of the MRS signal. Note that SV ^1^H MRS can detect the signal in specific regions of interest. The MRS spectrum revealed by the excitation is composed of peaks that represent the various hydrogen nuclei found in mobile molecules within the tissue. 

A metabolite may produce a number of characteristic spectral peaks, the pattern of which depends on the structure of the metabolite, magnetic field strength, and choice of MRS acquisition parameters, particularly the echo time of pulse sequences. The amplitude of a peak in the spectrum is directly proportional to the concentration of its corresponding metabolite, so that various metabolites can be distinguished and their levels quantified [20]. Evaluation of tissue metabolite composition by *in-vivo* MRS is complicated by the fact that the spectral lines are often broad – in consequence, peaks overlap, thereby decreasing resolution. For this reason, high-field MRS provides a fundamental benefit: the high magnetic fields increase the dispersion of uncoupled spins and multiplets, which leads to greater precision and accuracy of metabolite quantification, in turn leading to increased measurement reliability [21]. 

#### 3.1.2. Signal Localization, Spectrum Acquisition, and Water Suppression 

To achieve high spectral resolution and accurate metabolite quantification, it is often optimal to limit the signal source to within a localized voxel of interest (VOI) that has high magnetic field homogeneity. The MR signal is typically localized by sequentially applying three orthogonal gradient fields; each combined with a spatially selective radio frequency (RF) pulse [20]. The VOI resulting from the intersection of the orthogonal planes is typically a cube or rectangular prism with a volume ranging from 1-8 cm^3^. 

Several RF pulse sequences have gained popularity for VOI selection, including the Stimulated Echo Acquisition Mode Sequence (STEAM) [22, 23] and the Point Resolved Spectroscopy Sequence (PRESS) [24]. Shorter echo-times can be achieved using STEAM, but the method suffers from a factor-of-two loss in signal, compared to PRESS [25, 26]. PRESS is a double spin-echo technique that can be used with either longer or shorter echo times [23-26]. Note that spin-echo based sequences (*e.g.*, LASER - Localization by Adiabatic Selective Refocusing [27] and semi-LASER [28]) that utilize adiabatic slice selection have been used at high magnetic fields [29]. These techniques benefit from well-defined voxel profiles, despite higher power deposition and longer echo times [24, 27, 29-31]. At high fields, line-shape distortions caused by magnetic field inhomogeneities can be minimized by avoiding tissue-air boundary and implementing high-order magnetic field shimming within the VOI [32, 33]. 

Water molecules account for over three fourths of the mass and the water concentration of the brain, which is more than a 1000 times greater than most metabolites of interest. In consequence, neurometabolite signals can be identified only with water suppression [20], typically achieved by applying chemical shift selective (CHESS) pulses at the water frequency followed by crusher gradients to dephase the water signal immediately prior to the acquisition of the metabolite spectrum. This technique selectively suppresses the water signal, with very little effect on the signal intensity of other metabolites in the spectrum [34].

### 3.2. Quantification of MRS Metabolic Information

#### 3.2.1. Metabolite Estimation

Due to the large overlap of spectral peaks and peak distortion, fitting the spectrum often requires prior knowledge of peak line-shapes (*e.g.* Lorentzian), and accounting for the properties (*e.g.*, position, amplitude, line width, phase) representing each peak visible in the metabolite spectrum [35]. Following fitting, typically, the area under each fitted peak is calculated and the concentration of the metabolites is estimated in proportion to the area. Typically, levels of metabolites are adjusted for several additional VOI-specific confounders such as tissue partial volume (grey matter, white matter, cerebrospinal fluid) and tissue relaxation time constants [36]. This is of particular importance in studying AD, given the considerable atrophy rate in the patient population. Subsequently, standard statistical analyses of the levels of metabolites are performed. 

Levels of metabolites are usually reported in relation to a reference metabolite, with a known and stable concentration, measured in institutional units (IU) [20, 37]. The reference signal can be either external (*e.g.*, based on phantom replacement) or internal (*e.g.* the signal of water or other metabolites within the same VOI, *e.g.* Creatine and/or Choline) [38-40]. The approach of referencing to a metabolite is not without its critics, as the reference level can also vary [41, 42], particularly in relation to aging and dementia [43]. Ratios of metabolites quantified under identical conditions are often calculated to result in unit-free measures, which can be compared across experimental settings, whereas the quantification data in IU from different studies should not be directly compared [44]. 

#### 3.2.2. Software Specific for MRS Analysis

Several software packages have been developed specifically for MRS data quantification, including: (1) LCModel [45], widely used for automatic quantification of *in vivo* proton MR spectra, is a commercial software that accepts time-domain data input and generates one-page summary output through non-interactive, operator-independent analyses; (2) PROBE-Q [46], an on-line program embedded in the General Electric Medical System, displays and processes MRS spectra to provide measures of metabolite peak heights and ratios while they are acquired; (3) fitMANSuite [47, 48], a comprehensive MRS processing and qualification package in either time domain or frequency domain, has been used mostly in handling data acquired at high-fields using the LASER pulse sequence with a short TE; and (4) jMRUI [49], a distributed software package for time domain MRS and MRSI analysis that has a user-friendly graphical interface and uses a semi-parametric algorithm based on quantification of uncertainty in extreme scale computations.

### 3.3. Neurometabolites Quantifiable by *In Vivo *SV ^1^H MRS

#### 3.3.1. NAA, Cr, Cho, mI

Each of these neurometabolites has been studied using either low-medium or high fields [50-53]. *Acetyl Aspartate (NAA)*: *NAA* is an amino acid present almost exclusively in neurons and so is recognized as the most important chemical marker of neuronal density. A decrease in *NAA* concentration has been reported for several neurological disorders, likely reflecting a combination of neuronal loss, damage to neuronal structures, and/or reduced neural metabolism. The most prominent peak of *NAA* is at 2.01 ppm, while several smaller *NAA* peaks can overlap with glutamate and certain macromolecules. *Creatine (Cr)*: *Cr* is employed as an indicator of cellular energy state; *i.e.*, reserve for neuronal activities. The *Cr* resonance includes the signals from phosphorylated creatine and creatine, with a primary peak at 3.03 ppm and a second peak at 3.91 ppm. *Choline (Cho)*: The main *Cho* peak is located at 3.20 ppm, which includes signals from mobile choline compounds including free choline, glycerophosphorylcholine, and phosphorylcholine. An increase of *Cho* is considered a marker of pathological proliferation/degradation of cell membranes and demyelination, most commonly associated with neoplasms. An acute change in *Cho* level can also reflect changes in diet or medication. *Myo-Inositol (mI)*:* mI* is a polyalcohol that is present at high concentration in the glial cells, and is considered a glial cell marker. An increase in the *mI* level relative to NAA level has been linked with gliosis, to suggest regional neuronal damage. The *mI *spectrum contains four multiplets: the primary peak is at 3.57 ppm, the second major peak is at 4.07 ppm [50-53]. Compared with the above-noted three other major metabolites (*NAA*, *Cr*, *Cho*), *mI* is less reliably quantifiable, because of its strong overlap with peaks from several other metabolites.

#### 3.3.2. At High Fields

High field-strength increases SNR and spectral resolution [18, 19, 21]. Fig. (**1**) shows *in vitro* SV ^1^H MRS spectra at 1.5T and at 4.0T. At high fields, all metabolites show greater frequency dispersion, resulting in greater discrimination of peaks with similar chemical shift values, leading to more reliable quantification [48, 54-56]. For example, at 3.0T a peak at 3.91 ppm for creatine and at 3.93 ppm for phosphorylated creatine can be identified, while the mI peak can be resolved into peaks at 3.55 and 3.61 ppm. In addition, several metabolites that cannot be well quantified at low magnetic fields can be quantified at high fields, as detailed below. 

*Glutamate (Glu), Glutamine (Gln), γ-Aminobutyric Acid (GABA)*: *Glu* and *GABA* are the two most important neurotransmitters. *Glu* is an excitatory transmitter and a decrease in *Glu* may reflect the loss of glutamatergic neurons, or more greatly reduced synaptic function, or both. For this reason it should be complementary to *NAA* changes, but appears to have some potential to offer greater precision. *GABA* is an inhibitory neurotransmitter used to regulate activities of neurons and astrocytes, but its quantification in this setting has been problematic and will not be considered further here. *Gln* is a main precursor of both *Glu* and *GABA* [57]. The spectral peaks found between 2.04 and 2.35, and at 3.75 ppm are from *Glu*; between 2.12 and 2.46, and at 3.76, 6.82, and 7.73 ppm from *Gln*; and 1.29, 2.28, and 3.01ppm from *GABA *are heavily overlapped, but quantification of these metabolites is possible at high fields [21, 58, 59]. *Glucose (Glc)*: *Glc* is the primary energy source of the neurons. The substance has a complex multiplet spectrum at 3.44, 3.81, and 5.23ppm [60, 61]. *Glutathione (GSH)*: *GSH* is present in all types of human cells, with high concentrations in major organs such as the brain. Its peaks are located at 2.15, 2.55, 2.93, 2.98, 3.77, and 4.56 ppm [62, 63]. The physiological functions of *GSH* include detoxification of harmful reactive oxygen species generated during different molecular processes and is considered a “repair” marker. *N-acetyl aspartylglutamate (NAAG)*: *NAAG* is a neurotransmitter that modulates glutamatergic neurotransmission [64]. The largest resonance of *NAAG* is at 2.04 ppm, but excellent magnetic field homogeneity is required to separate *NAAG* from *NAA* [65], *i.e.*, the VOI must be shimmed precisely. Several neurologic diseases involve specific *NAAG* changes, and greater interest in understanding the role of *NAAG* is emerging. *Scyllo-Inositol (sI)*: *sI* is a product of *mI* metabolism and can act as a stabilizing isomer to prevent the formation of neural toxic substances [66]. This chemical has a structure similar to the *mI* with a single peak at 3.34 ppm [67, 68]. *Lactate (Lac)*: *Lac* has a doublelet at 1.31 ppm, with another peak at 4.01ppm. The substance is a product of anaerobic glycolysis metabolism, typically detectable in brain diseases under hypoxia conditions (*e.g.*, stroke, encephalopathy) [69, 70]. Finally, *Taurine (Tau)*: *Tau *is associated with two sets of spectral peaks at 3.25 ppm and 3.42 ppm [71, 72]. This highly abundant organic acid activates *GABA* functions and has a role in neuronal protection and cerebral volume regulation [73, 74].

## 4. RESULTS 

### 4.1. MRS-Based Studies on Metabolite Profiles in AD at Low Fields

A large number of studies on AD using low-fields SV ^1^H MRS published by 2007 have been largely reviewed by Kantarci and others [14, 75], although since then over 30 additional low-field studies have been published. These are briefly summarized below (Table **1**). 

In general, a decrease of *NAA*, reported in the medial temporal lobe (MTL), posterior cingulate gyrus (PCG) and virtually each major cortical lobe, represents the most robust MRS finding in probable/possible AD, likely reflecting disease-related neuronal loss/dysfunction. A decrease in *NAA* and an increase in *mI* have both been detected prior to evident medial temporal lobe atrophy [76]. Even so, they may not always be found simultaneously in the same VOIs, leading to questions about reliability at low fields [77-80]. Changes of *Cr* and *Cho* in AD also appear to be less consistent using low fields [81-88]. It appears that the inconsistent *Cho* results can be attributed sometimes to differences in VOI placement and variable intake of choline-containing food and medication across studies [89, 90]. 

Longitudinal MRS investigations of at-risk people (*e.g.*, MCI) that involve multiple time-point MRS scans are especially important in understanding disease progression. In the few such studies available, subjects with MCI have been followed for 1-3 years, to characterize metabolite profiles of those who converted to dementia (Table **1**). In general, compared to non-converters, MCI to AD converters show greater reductions in *NAA* or *NAA/Cr* in different cerebral locations between baseline and follow-up [91-96]. *NAA* or *NAA/Cr*, especially those of the hippocampus, often correlate with memory test scores [83, 92, 97, 98] and so can be related to memory function and to predict AD with relatively high accuracy [91, 92, 98]. They also showed some localization effects, including two distinct metabolite profiles: whereas most subjects with MCI displayed a decrease in *NAA*, *Cho*, *Cr*, and *Glx* at the one-year follow-up, 36% of subjects showed an increase in *NAA*, which was associated with an improvement of executive function [82], suggesting a neurocompensatory response early in the course of AD. A study by Schott *et al.* (2010), which involved six MRS evaluations over 24 months and compared clinically confirmed AD with healthy controls, reported that the baseline *NAA/mI* in the posterior cingulate gyrus distinguished AD from HC, with approximately 80% sensitivity and specificity [99]. 

### 4.2. MRS on AD Studies at High Fields

#### 4.2.1. Metabolite Profiles in AD, MCI, and Aging

Table **2** provides a list of the *in vivo *SV ^1^H MRS studies in AD using high-field MRI systems (*e.g.*, 3.0T or 4.0T). These high-field studies have reported quantitative metabolite levels and/or their ratios, which have largely verified AD-characteristic *NAA* decrease and *mI *increase, but with greater reliability of metabolite quantification. A few other metabolites have also been quantified, frequently differed between AD, MCI, and healthy aging (Table **2**). Each of the high-field studies employed 3.0T unless specified otherwise.

Hattori *et al.* (2002) reported a correlation between the reduction of *NAA/Cr* and (*Glu*+*Gln*)/*Cr* in the posterior cingulate region in AD [100]. Kantarci and others (2003) compared the MRS profiles at 1.5T and 3.0T in the posterior cingulate VOI, by enrolling a relatively large sample of subjects with AD, MCI, and normal aging [101]. The study reported an increased signal to noise ratio and a greater spectral resolution at the higher field, leading to more consistent measures with *Gln*/*Cr*, (*Glu*+*Gln*)/*Cr.* However, based solely on *NAA*/*mI*, the quantitative gain did not translate to enhanced AD and MCI discrimination [101]. Rupsingh *et al.* (2009) conducted a 4.0T study to investigate MRS profiles in subjects with AD and MCI, and in matched controls. Data were acquired from a VOI placed in the right hippocampal region. Confirming a significantly lower *NAA/Cr* in AD than in HC, the study also reported a quantitatively lower level of *Glu/mI* in AD than in MCI, possibly reflecting its greater sensitivity than *NAA/Cr* (that did not show a significant difference) [102]. 

Griffith *et al.* (2010) examined group differences in *NAA/Cr*, *mI/Cr*, and *Cho/Cr* between subjects with MCI and healthy aging in the posterior cingulate gyrus. An average increase of *mI/Cr* and *Cho/Cr* was reported in MCI in contrast to HC, whereas no difference in *NAA*/*Cr* was found [103]. Of note, the group also reported significant correlations between the executive function and the level of *NAA/Cr* (positively) and *mI/Cr* (negatively) in the posterior cingulate gyrus in MCI [104, 105]. In the study by Lim and colleagues (2012a), a close relationship was found between the verbal memory testing scores and the level of *NAA/Cr* in each of the six VOIs covering the posterior cingulate gyrus and the surrounding regions [106]. Lim *et al.* (2012b) also investigated *NAA* and *mI* profiles in the anterior and posterior cingulate gyri for AD, MCI, and HC. They reported decreased *NAA/Cr* of the posterior cingulate gyrus and increased *mI/Cr *of the anterior cingulate gyrus in relation to cognitive testing scores, corresponding to the posterior-dominant progression of AD pathology [107].

Kaiser and colleagues (2005) used 4.0 T MRI, to study differences in the corona radiata white matter between older and younger adults [108]. The study reported an elevation of *sI* in older adults compared to younger adults, which paralleled the changes of *mI* and *NAA*. This study showed that neurochemical changes in the aging brain might be reflected also by *sI*, in addition to *NAA* and *mI*, even though the latter were more reliably quantifiable [108]. Griffth *et al.* (2007) published the first study investigating MRS-based *sI* changes in patients with amnestic MCI and mild AD [109]. In the posterior cingulate gyrus, patients with either mild AD or MCI showed an increase in *mI*/*Cr *compared to healthy aging. A decrease in *NAA*/*Cr* was also found, but only in AD. The *sI*/*Cr* also increased in AD and correlated negatively with cognitive performance.

The 4.0T MRS study by Emir and colleagues (2011) marked the first *in vivo* report of a lower level of *GSH* in aging in the occipital lobe voxel, which was accompanied by a higher level of *Lac* [110]. In the following year, Mandal *et al.* (2012) used participants with MCI and AD in a *GSH* focused study*,* and reported significant *GSH* reduction in AD, but less clearly so in individuals with MCI [111]. These studies however are useful in providing at least initial insights about the role of MRS-based *GSH* quantification in cognitive impairment. In addition, an elevation of posterior cingulate gyrus *Lac *was correlated with poor memory performance [112]. Weaver *et al.* (2010) tested amnestic MCI patients using a VOI placed in the posterior cingulate gyrus. The association between cognitive decline with an increase in *Lac* and with a decrease in *GSH* provides new evidence that in addition to neuronal damage (as revealed by *NAA* and *mI* changes) pathological aging likely involves an anaerobic process and reduction in anti-oxidation effectiveness. 

#### 4.2.2. Regional Metabolite Differences in AD

Reflecting that brain regions are differentially affected in AD [2], regional metabolite profiles have shown stage-dependent spatial differences [11]. Applying multiple single VOIs, differences between HC and MCI were found most often in those regions affected earliest (Table **2**). Seo *et al.* (2012) reported a study comparing amnestic MCI and HC with as many as four single voxels, placed in the left entorhinal cortex, left hippocampus, posterior cingulate gyrus, and the occipital white matter [113]. The study identified a group difference in *NAA*/*Cr* between MCI and HC only in the entorhinal VOI, possibly reflecting the very early involvement of this brain region in cognitive changes. 

The hippocampus and posterior cingulate gyrus have been most commonly studied; their metabolite profiles are not identical. Some left-right hippocampal differences were also reported. For example, even though a reduction of *NAA*/*Cr* in both left and right hippocampi was seen in subjects with mild AD, such a reduction was detected only in the right hippocampus in people with mild memory impairment [114]. Wang *et al.* (2012) also noted differences between left and right hippocampus. In their study, subjects with amnestic MCI showed significantly increased *mI/Cr* in the left, but not the right hippocampus and the* NAA/mI* in the posterior cingulate gyrus best separated amnestic MCI from HC subjects [115]. Another study comparing the hippocampus and the posterior cingulate gyrus in subjects with AD, MCI, and HC suggested that decreases in *NAA*/*Cr* and increases in *mI*/*Cr* and *mI*/*NAA* were more significant in the hippocampus. The increase in *mI* occurred early in MCI, which could be used to distinguish between HC and MCI, but not between MCI and AD [116]. In 2013, Bittner and colleagues reported that the hippocampal *NAA/Cr* could be used to identify AD from HC with high sensitivity (94%) and specificity (92%) [117]. In addition, they reported significant associations between MRS based *NAA/Cr* quantification and the cerebrospinal fluid (CSF) biomarkers [117]. 

Mihara and others (2006) compared subjects with AD, frontotemporal dementia, and healthy aging using four single voxels placed respectively in the anterior and posterior cingulate gyri, prefrontal white matter, and the parieto-occipital white matter [118]. The study verified posterior-dominate metabolite changes in AD, with more pronounced *NAA*/*Cr* reduction in the posterior cingulate gyrus and the precuneus regions than in the frontal regions, and a low posterior to anterior ratio for *NAA*, which was used to differentiate AD from frontotemporal dementia [118]. 

#### 4.2.3. Treatment Evaluation

As with low-field studies, high-field MRS work on treatment has typically involved only relatively small groups of patients, with MRS tests before and after treatment. In many cases, if subjects with normal aging had been recruited for comparison, they were only scanned at baseline. MRS data demonstrate just modest responsiveness with either ChEI or memantine (Tables **1**, **2**)_._ Earlier studies have suggested a similar spectroscopic effect of donepezil and memantine on mild to moderate AD, when the two medications were administrated separately [119].

Henigsberg and others (2011) reported a post-treatment increase of *NAA*/*Cr* in 10 of 12 people with mild-moderate AD treated with donepezil; the voxel was placed in the left dorsal lateral prefrontal cortex (regarded as the centre of executive function), which was felt likely to respond to ChEI treatment [120]. The finding is intriguing, as it can imply a possible treatment-related neurocompensatory enhancement in this brain region. Unfortunately, the SNR of the metabolite measurement data appeared to differ between pre and post treatment scans [120]; further investigations will need to address the robustness of this finding. 

*Glu* (involved in glucose metabolism) is generally considered to be more sensitive to ChEI treatment and therefore better responsiveness is expected with *Glu* than with NNA. 

**Fig. (2):**
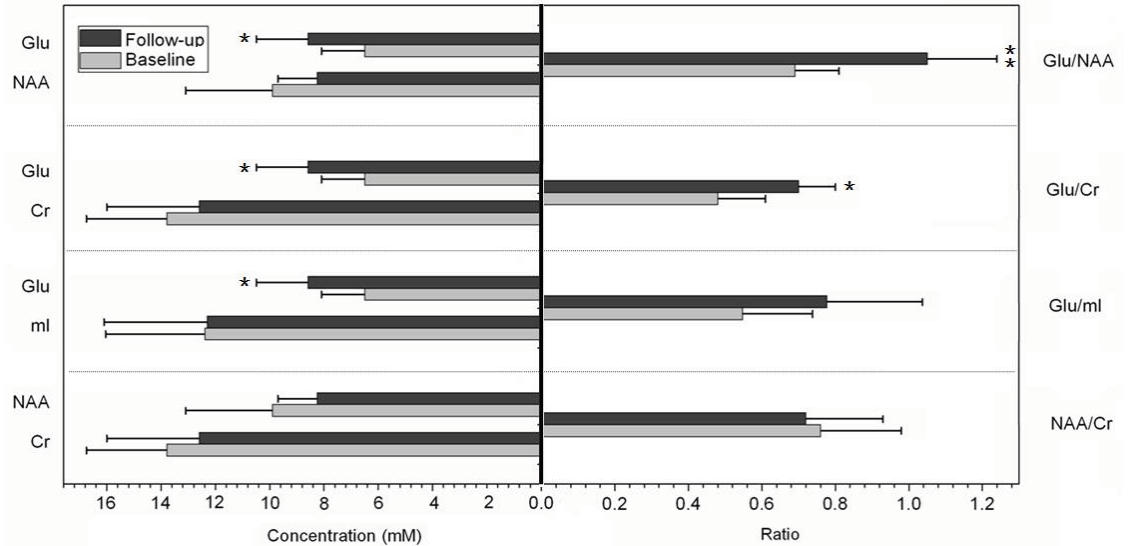
Changes in the level of metabolites in the right hippocampal VOI following galantmine treatment. An increase in glutamate concentration and ratio after treatment in patients with Alzheimer’s disease was observed. (data were retrieved from Penner *et al.*, 2010 [122])

Indeed, a number of investigations have reported significant post-treatment effects using MRS-based *Glu* quantification. Bartha and colleagues (2008) scanned 10 subjects with mild AD at baseline and following four months of donepezil treatment [121]. A decrease in levels of *NAA*, *Cho*, and *mI*/*Cr* were observed after treatment, whereas the level of *Glu* remained unchanged over time, suggesting a positive effect of the medication [121]. In a later study by the same group, an increase in *Glu* was reported following four months of galantamine treatment in patients with AD (Fig. **2**) [122]. The increase of *Glu* was accompanied by a marginal decrease of *NAA* over time, suggesting independence of the cholinergic and hippocampal degenerative mechanisms [122].

Glodzik and colleagues (2008) [123] conducted a study to investigate the effect of memantine treatment (which presumably modulates the glutamate-induced excite-toxicity in AD by stabilizing the NMDA receptors). The study was conducted using 3.0T MRS in the bilateral hippocampal region. Metabolite data were collected in both cognitively impaired and cognitively normal individuals at baseline and 6-month follow-up, between which patients with AD, MCI, and a subgroup of older controls received stabilized memantine treatment for 20-24 weeks. The study reported a reduced rate of change for *Glu*/*Cr* in the left hippocampal region in the treated compared to the non-treated subjects, while a change in *Glu/Cr* was not found in the right hippocampal region, nor for *NAA*/*Cr* in any other regions studied [123]. 

Ashford *et al.* (2011) reported a pilot double-blind placebo controlled study to test the possible effect of memantine in treating patients with AD [124]. Seven patients received the medication and the other six patients a placebo agent. MRS data were collected from VOIs located in the posterior cingulate gyrus and the left inferior parietal lobe. At the pilot stage, this study failed to detect a treatment-induced change in subjects with mild-moderate AD, based on either the *NAA*/*Cr* metabolite ratio, or the cognitive test scores [124]. Gordon *et al.* (2012) aimed to characterize disease progression acquired MRS spectrum from the precuneus-posterior cingulate area. The authors noted an increased *mI *and a decreased *NAA* in mild to moderate AD patients following treatment with a ChEI (either donepezil or galantamine) and memantine, but found no other metabolite changes or cognitive decline [125]. 

In a 2013 sub-study of a randomized, double-blind, placebo-controlled clinical trial published in *JAMA Neurol*, Friedman *et al.* reported the effect of investigation with growth hormone–releasing hormone on several metabolites including *GABA*, *Glu*, and *NAAG* 20 weeks post-treatment in subjects with MCI and healthy aging [126]. MRS data were acquired from three VOIs loaded in the posterior cingulate, dorsolateral frontal, and posterior parietal regions of the left hemisphere. Increased *GABA* levels were detected in all the three VOIs, together with increased *NAAG* in the frontal regions and decreased *mI* in the posterior cingulate gyrus. The study provided initial evidence on favorable effects of the treatment on aging with modulation of a major inhibitory neurotransmitter in the brain.

#### 4.2.4. Combining Multiple MRS Metabolites

As multiple metabolites and/or their ratios can show a traceable difference between diagnostic groups, even when each of them (when considered individually) may not necessarily be significant statistically, several papers have explored the possibility of combining data from multiple metabolites. *NAA* and *Glu* and their ratios to *Cr* have been used most often. For example, examined using levels of multiple metabolites within the same hippocampal VOI, Rupsingh *et al. *(2011) found that the accuracy of discriminating clinical AD from HC was 71% using *NAA*/*Cr*, 80% using *Glu*, and 94% using a combination of the quantitative *NAA*/*Cr*, *Glu*, and *mI* [102]. Similarly, in the study by Wang and others (2009), the increased *mI*/*NAA* in AD in both hippocampal and posterior cingulate VOIs was used for AD and HC classification; a better performance was obtained than when using data from a single VOI [116], as also reported by Azevedo and colleagues (2008) using a lower field [127]. These observations linked the uneven spatial expression to progressive distortion of brain structures compromised by the disease [11], suggesting some potential of MRS-based evaluation of multiple neurochemicals, and of combining metabolite data acquired from more than one brain region.

Perhaps an even greater potential exists in discriminating MCI from AD by combining quantitative MRS with other neuroimaging methods (*e.g.*, structural MRI based medial temporal lobe atrophy and cortical thickness), as well as with clinical test data (recognizing their relative insensitivity compared with MRS). A number of low-field studies that combined MRS with neuroimaging methods (*e.g.*, hippocampal volume and white matter hyperintensity [128], diffusion tensor imaging [129], MRI-based volumetric cortical thickness [130]) have also reported high sensitivity and specificity in the discrimination of AD from MCI. In addition, some low-field studies report value in combining MRS and other neuroimaging methods to predict which high-risk individuals might convert to AD-dementia [93, 131]. The potential of high-field MRS in this regard is yet to be explored.

In a study linking PET-based A*β* biomarker and MRS-based neurochemical measurements, Kantarci *et al.* (2011) studied cognitively normal older subjects and showed an association between higher PiB retention (a specific label for fibrillary A*β*) with an abnormal increase of the *mI*/*Cr* and *Cho*/*Cr* ratios [132]. Meanwhile, higher *Cho*/*Cr* was associated with worse domain-specific cognitive performance, which was independent of A*β* load, suggesting the involvement of additional pathologies [132]. Indeed, disease related metabolite changes in the white matter have been suggested by a number of studies [87, 113, 121, 132], largely reflecting their involvement in both vascular cognitive impairment and AD [133-135]. 

## 5. DISCUSSION

### 5.1. Summary 

The development of high-field single-voxel ^1^H MRS technology has enabled *in vivo* measurement of brain metabolites that cannot be reliably quantified at lower magnetic fields. The spectrum has greater chemical shift dispersion and sharper associated peaks at high fields, allowing more reliable metabolite quantification. Such technological enhancements can allow better understanding. In total, 27 high-field (3.0T or 4.0T) MRS research papers on AD have been published, of which 23 have appeared since 2008, marking the progress of the field (Table **2**). 

Most high-field studies have investigated the differences in brain metabolites (and/or their ratios) between early AD (and MCI) and HC. Two studies have attempted to differentiate AD from other dementias (*e.g.*, Parkinson's disease and frontotemporal dementia) [105, 118]. Three studies have focused on aging and dementia risks [108, 110, 132]. More than half of these studies involved people at early stages (*e.g.*, MCI or subjective memory complaint). Three studies have investigated treatment effects with ChEI agents – donepezil [120, 121] or galantamine [122]-while two other studies examined the effect of memantine, an uncompetitive NMDA (N-methyl-D-aspartate) receptor antagonist based on brain metabolite data [123, 124], and one tested ChEI and memantine treatments in combination [125]. Regarding the voxel location in the high field investigations, the posterior cingulate gyrus region was studied in 70% of the investigations, followed by the hippocampal structure and surrounding regions (33%, despite notable technical difficulties, related to increased susceptibility to signal degradation at high fields). Other brain structures examined included the prefrontal and the anterior cortical regions, parietal cortex (26%), posterior and anterior deep white matter (15%), and the temporoparietal, and occipitoparietal regions (<10%; Table **2**). 

**Fig. (3):**
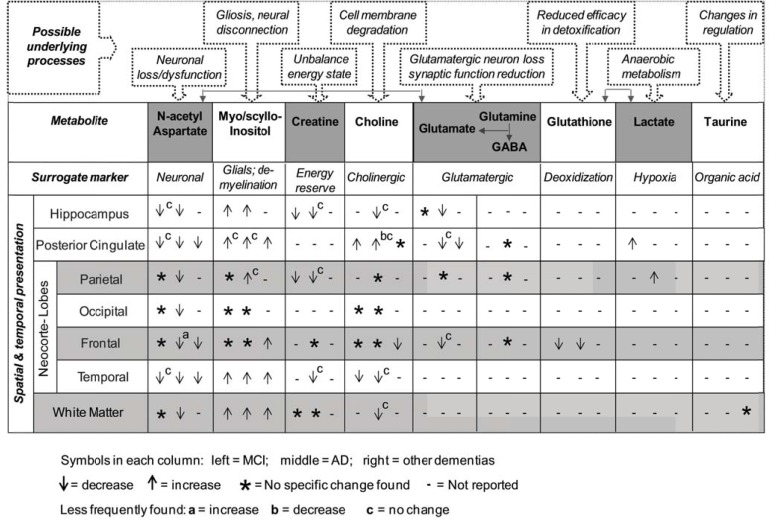
Sketched diagram illustrating featured spatial and temporal patterns of metabolites changes detected by ^1^H MRS at high-fields, linked to possible underlying molecular/cellular processes.

The high-field studies have generally verified the characteristic neurochemical changes that are documented for clinical AD at low fields, but with more reliable and robust quantification, albeit with less data from the hippocampus and enterorhinal cortex. As a surrogate neuronal marker of AD, *NAA* has been studied most frequently, with a consistent reduction of *NAA* (or *NAA/Cr*, *NAA/mI*) in each of the major cortical lobes in established AD [136]. Similar changes with *mI* likewise have been reported consistently. In addition, decreased *Glu* levels, possible only with high-field MRS, have been found not just to accompany decreases in *NAA*, but typically to be more sensitive to AD changes [121]. In addition, high-field findings have suggested that several additional metabolites might also be quantifiable, with group differences between subjects with AD and healthy aging (Fig. **3**). Such changes may reflect an in-sync involvement of more heterogeneous mechanisms in the disease expression (*e.g.*, oxidative, inflammatory, and vascular components) [132, 137]. 

Because a large portion of people with MCI progress to AD, many reports note that investigating changes at the MCI stage might benefit early AD detection. This should be considered with caution: people with MCI can have highly variable profiles not just clinically, but in disease pathology and progression, and overlap with AD and HC is common [138]. What makes MRS promising compared to other AD biomarker approaches being tested? First, MRS provides a direct quantification of the concentration of several important neurometabolites, in contrast to more qualitative descriptions seen with most functional neuroimaging measures (*e.g.* functional MRI and FDG-PET) [128-130, 132]. In this regard, emerging evidence suggests that levels of key metabolites (*e.g.* *NAA*) are correlated with amyloid imaging; the latter requires administration of radioactive agents - Pittsburgh compound B or ^18^F-Florbetaben [139], as well as with the CSF A*β*-42 and tau protein biomarkers; the latter do not address spatial presentation [132]. In addition, changes in neurochemicals have been related to Braak staging patterns [2, 15, 16, 140]. Moreover, by evaluating metabolites of multiple neurochemicals, light can be shed on the heterogeneous pathologies of MCI and AD instead of focusing merely on the A*β* pathology. MRS also has potential to allow for more early differentiation of the diagnosis of AD from other dementias based on spatial location information (Fig. **3**). Compared to volumetric morphology of the hippocampus and the evaluation of cortical loss, which are accepted aids in AD diagnosis, MRS-based metabolic changes can be detected much earlier than atrophy based structural brain changes [76]. 

### 5.2. Future Directions 

Given the wide range of brain structures and processes involved in AD, integrating multiple sources of information about disease progression is useful [141]: this same line of reasoning can be extended to MRS information about multiple metabolites. To do so would be an advance. Most MRS studies to date typically have focused on detecting changes in individual metabolites or their ratios. Such a “one thing at a time” approach appears to be insufficient in dealing with a complex problem. The data under review suggest that changes in metabolites between diagnoses or in response to treatment can be small and insignificant statistically, when considered individually. In consequence, these changes often have not been integrated into useful information (Fig. **3**). 

Adding to this challenge is that metabolite changes are often not universal across brain regions, but spatially heterogeneous, related to the variable regional effects of the disease [2, 11, 112, 142]. As a result, studies with otherwise similar approaches can reach different conclusions when spectra are acquired from different regions (Fig. **3**). To further complicate the problem, brain metabolite changes continue throughout the disease process [143, 144]. For example, changes in *NAA* and *mI *between HC and AD/MCI have been detected in the MTL and the posterior cingulate gyrus, whereas no such differences are shown in other brain regions that are affected later in the development of AD [113, 116].

We argue that to effectively characterize AD-associated neurochemical changes using MRS, data on multiple quantifiable metabolites from different brain regions should be analyzed interactively. Indeed, combining metabolite data from different VOIs can improve AD identification [116], so too with combining information of multiple metabolites [102, 127, 136]. The performance of MRS can be further improved when data are combined with multimodal neuroimaging data and clinical assessment data [128-130]. Here, data-driven computational techniques hold obvious promise. Even though MRS data mining and knowledge discovery have yet to become popular, such methods have proven useful in exploring health data as well as serum NMR data [145, 146]. Importantly, if MRS is to realize its long awaited potential, the field will need to embrace standardization and accessibility. Therefore, attention should be given to improving the generalization of exploratory methods in MRS research, such as by applying independent datasets for validation when a model is derived.

From a technical viewpoint, MRS involves strict requirements for both spectrum acquisition and processing. Analysis must be individualized to the VOI and as such is time consuming, and even with the higher level of automation, accurate quantification can be a concern. Currently, MRS is not regarded as robust a method as are volumetric MRI measures of the medial temporal lobe atrophy [3, 6, 15, 16]. Even so, with standardized reliability and validation assessments, MRS can provide a more reliable method in quantifying functional brain alternations, to provide additional information of AD. It is particularly valuable at early stages prior to the presence of detectable morphometric changes. 

In this regard, high-field systems can result in improved predictive power. For example, a 20% improvement in sensitivity could be realized at 3.0T compared to 1.5T [54]. A profile with up to 18 neurochemicals (including alanine, aspartate, ascorbate/vitamin C, N-acetylaspartylglutamate) can be quantified with *in vivo* ultra-high field MRS (*e.g.* 7.0T) in humans, even from small brain structures (*e.g.* substantial nigra [110, 147]. Of note, one of the more intriguing developments to emerge from the large shadow long cast by beta-amyloid has been the understanding of how the AD brain metabolizes glucose. Various “Type III diabetes mellitus” or “starving in the face of plenty” metaphors have been proposed to indicate deficiencies in glucose transport, as well as compensatory mechanisms [148, 149]. Such work may benefit from MRS investigations. Although as yet data are sparse, its potential future role is anticipated. Clearly, the fundamental solution to realize MRS as a diagnostic tool lies in continuous technology advancement. 

Finally, to demonstrate the value to clinical evaluation and management of dementia, MRS findings from longitudinal studies are of fundamental importance. Currently, the number of high-fields longitudinal investigations is limited. The majority of published follow-up studies assessing treatment effect at high-field have typically employed only a small sample size, often without controls; too often the use of medications that might be relevant has not been reported. These caveats must be addressed to allow MRS to serve as a neuroimaging marker with many potential uses, including: clinical diagnosis and prognosis, progression tracking, treatment evaluation, and novel therapy development. 

## CONCLUSION

The measurement of changes in multiple neurochemicals in localized brain regions by SV ^1^H MRS is not restricted to measuring increases or decreases, but involves accurate quantifications. In this way, it provides valuable information linking neural chemical alterations to neuropathological deficits and early cognitive decline, beyond those that can be offered by other neuroimaging techniques. High-field MRS has significant advantages in increased signal to noise ratio and spectrum resolution, leading to greater quantification reliability, as well as the detection of a greater range of neurometabolites. Increased signal to noise ratio at high fields can also be traded for the use of smaller voxels with enhanced anatomical consistency and tissue uniformity, further improving quantification. 

High-field MRS holds promise to reveal patterned metabolite profiles of AD. Further studies will need to enhance understanding of the pathological basis of the MRS findings and to translate the advantages of high-field findings to enhanced diagnostic decision-making. The use of MRS in sub-studies of controlled trials, as recently seen [126], has much to recommend in this regard. Standardization of data acquisition and processing can help realize its full potentials in multi-centre studies, especially with regard to: (1) longitudinal changes in metabolite profiles and their association with the outcome of people at high-risk for AD; (2) metabolite changes in response to treatment involving larger samples of AD and matched controls; and (3) early AD diagnosis in combination with multimodality neuroimaging and clinical assessments. 

## Figures and Tables

**Fig. (1):**
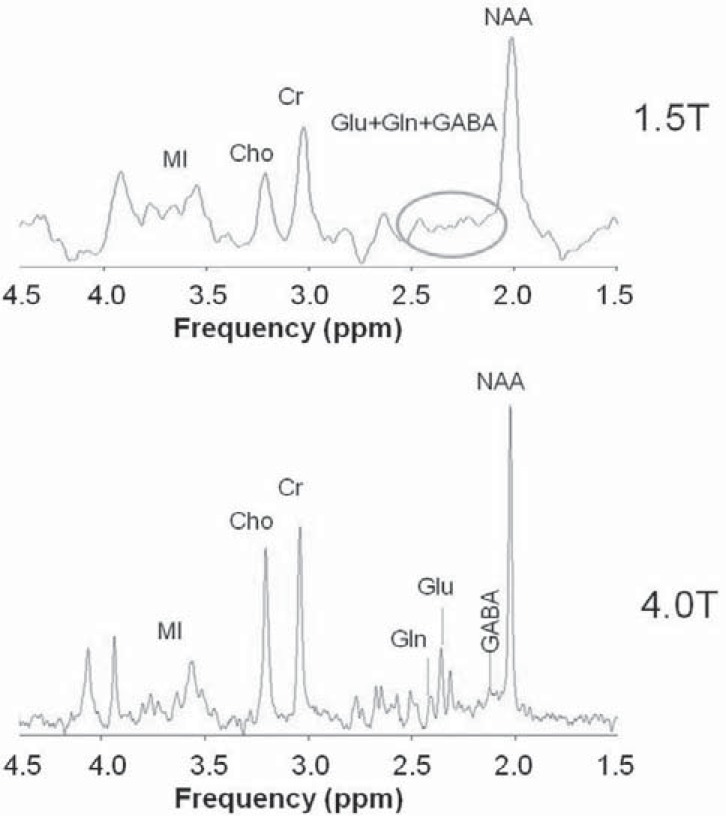
A comparison of the single voxel proton magnetic resonance spectroscopy spectra acquired at 1.5 T and at 4.0 T *in vitro.* Increase signal to noise ratio and spectral resolution were observed at 4.0T.

**Table 1.** *In vivo* single voxel proton magnetic resonance spectroscopy studies on Alzheimer’s disease at low field (<1.5 T) since 2007.

First Author	Year	Subject	Metabolites	Locate	Methods	Main Findings
Rami L	2008	AD=27 (mild); FTD=12; aMCI=30; HC=26	NAA/Cr, Cho/Cr	2 VOIs: temporal pole; left temporoparietal lobe	1.5T, PRESS, short TE (TR/TE= 1500/35ms)	1) Language performance related with NAA/Cr and Cho/Cr (r>0.331, p<0.005) in left temporal pole; 2) no correlation in the left temporoparietal.
Ding B	2008	AD=34 (mild=17, severe-moderate =20); HC=20	NAA/Cr, Cho/Cr, mI/Cr	posterior cingulate gyri (PCG)	1.5T, PRESS, short TE (TR/TE= 1500/35ms); DTI	1) Higher mI/Cr in mild AD than HC; 2) lower NAA/Cr in AD than in HC, particularly in moderate-severe AD; 3) mI/Cr positively correlated with left-side FA value in mild AD (r=0.524, p=0.037); 4) NAA/Cr negatively correlated with right-side MD in moderate to severe AD (r=-0.589, p=0.008); 5) NAA/Cr related with left-side FA and left-side MD in HC (r=0.542, -0.465, p<0.045).
Azevedo D	2008	AD=13; CIND=12; HC=15	NAA, Cho, Cr, mI; NAA/Cr, Cho/Cr, mI/Cr	3 VOIs: right temporal; left parietal; midline occipital	1.5T, short TE (PRESS, TR/TE= 2000/35ms for R temporal; STEAM, TR/TE=2000/30ms for other two regions)	1) No metabolite differences between AD and HC in any brain region; 2) higher parietal Cho in HC than in CIND; 3) identify AD from HC using (temporal mI + parietal mI) with accuracy/sensitivity/specificity of 75%/69.2%/80%; 4) identify AD from HC using (temporal mI + parietal mI + temporal NAA/Cr) with 85.7%/92.3%/80%; 5) identify CIND from HC using parietal Cho with accuracy/sensitivity/specificity of 81.5%/75%/86.7%.
Kantarci K	2008a	n=54 (from normal to AD)	NAA/Cr, Cho/Cr, mI/Cr, NAA/mI	PCG	1.5T, PRESS, short TE (TR/TE=2000/30 ms); linked with postmortem pathology	1) Decreases in NAA/Cr and increases in mI/Cr correlated with increasing severity AD-type pathology at autopsy; 2) metabolite related with pathology measurement (r2>0.40, p<0.001), NAA/mI most strongly related to Braak stage (r2=0.47, p<0.001).
Garcia Santos JM	2008	mild to moderate dementia=12 (AD=6, VaD=3, AD/VaD=3); MCI=10; HC=34	NAA/Cr; mI/Cr; Cho/Cr, NAA/mI, NAA/Cho	PCG	1.5T, PRESS, long and short TE (TR=1500 ms, TE=144/35 ms)	1) Lower NAA/Cr and NAA/Cho in AD than in MCI or HC; 2) NAA/Cr (TE=35 ms, cutpoint=1.4) distinguishing AD from HC with accuracy/sensitivity/ specificity of 0.83/91.2%/77.8%; 3) MCI can not be distinguished from HC by using any metabolite.
Olson BL	2008	MCI=47; HC=24	NAA, Cho, Cr, mI, Glx; NAA/Cr, NAA/Cho, Cho/Cr, mI/Cr	PCG	1.5T, STEAM, short TE (TR/TE/TM= 2000/30/13 ms); longitudinal, MRI scan interval: 11.56±4.3 months	1) Baseline: lower NAA, NAA/Cho, NAA/mI and higher Cho/Cr, mI/Cr in MCI than in HC; 2) over time: increased NAA, Cr, Cho, mI and decreased WM in 36% atypical MCI; decreased NAA, Cr, Cho, Glx in 64% typical MCI; decreased NAA/Cr and increased mI/Cr in HC; 4) in atMCI: NAA, Cr, mI and Glx negatively correlated with executive function; 5) NAA/mI and mI/Cr negatively related to executive function in MCI.
Watanabe T	2008	AD=30 (mild -moderate); Binswanger's disease=13; HC=26	MAA, mI; NAA/Cr, Cho/Cr, mI/Cr, NAA/mI	8 VOIs: L+R Hipp; anterior & posterior periventricular, deep WM; PCG; occipital	1.5T, PRESS, short TE (TR/TE=2000/30 ms)	1) Lower NAA in AD than in HC in most of the brain regions except for PCG; 2) lower NAA/Cr in most of the brain regions except for anterior PDWM; 3) lower NAA/mI in AD than in HC in all brain regions; 4) higher mI in AD than in HC in PCG; 5) higher mI/Cr in AD than in HC in hippocampus and R posterior PDWM; 6) at 80% specificity, hippocampus NAA identify AD from HC with sensitivity of 100%; and NAA/mI of 87%, and NAA/Cr of 77%.
Thambisetty M	2008	AD=13 (mild -moderate); aMCI=13	NAA/mI	hippocampus	1.5T, PRESS, short TE (TR/TE =1500/35 ms)	Hippocampal NAA/mI positively correlated with AD-specific plasma biomarkers in AD (r>0.6, p≤0.05).
Fayed N	2008	aMCI=119	NAA/Cr, Cho/Cr, mI/Cr, NAA/mI, NAA/Cho, mI/NAA	2 VOIs: PCG, left occipital cortex (LOC)	1.5T, PRESS, short TE (TR/TE=2500/30 ms); clinical follow-up; 29 month (convert to 49 AD, 5 Lewy Body Dementia (LBD), 28 MCI, 15vascular MCI (VaMCI), 22 depression with MCI (DeMCI)	1) PCG: NAA/Cr differentiate AD with DeMCI and MCI and VaMCI; NAA/mI differentiate AD with DeMCI and MCI; MAA/Cho differentiate AD with DeMCI; 2) LOC: NAA/Cr differentiate AD with DeMCI and MCI and VaMCI; NAA/mI differentiate AD with DeMCI; Cho/Cr differentiate AD with MCI; mI/NAA differentiate AD with DeMCI and MCI; 2) the best prediction of MCI-AD conversion: NAA/Cr<1.40 with accuracy/sensitivity/specificity of 82%/82%/72% in PCG, and NAA/Cr<1.57 with 79%/78%/69% in LOC.
Kantarci K	2008b	aMCI=32; naMCI=20; single-domain aMCI=91; multi-domain HC=100	NAA/Cr, Cho/Cr, mI/Cr	PCG	1.5T, PRESS, short TE (TR/TE=2000/30 ms)	1) Smaller hippcampus volumes and higher mI/Cr in single-domain aMCI than in naMCI and HC; 2) naMCI has normal hippcampus volumes and metabolite; 3) the majority of naMCI (15%) showed cortical infarctions compared to single-domain aMCI (7%).
Zhang B	2009	AD=13 (mild); MCI=9; HC=13	NAA/Cr, mI/Cr	2 VOIs: Hipp (L), temporoparietal WM (TPWM)	1.5T, proton regional imaging of metabolites (PRIME) sequence, short TE(TR/TE= 2000/25 ms); ADC value in DWI	1) Decreased hippocampal NAA/Cr in AD than in MCI or HC; lower TPWM NAA/Cr in AD than in HC; higher Hipp mI/Cr in AD and MCI than in HC; 2) at 84.6%specificity, the sensitivity for AD/HC=76.9% by Hipp NAA/Cr, 92.3% by mI/Cr; 100% by NAA/Cr+mI/Cr+ ADC; 3) at 84.6% specificity, the sensitivity for MCI/HC=21.4% by Hipp NAA/Cr, 78.6% by mI/Cr, 92.9% by NAA/Cr+mI/Cr+ADC; 4) NAA/Cr (r=0.58) and mI/Cr (r=-0.51) correlated with cognitive test (p<0.01).
Siger M	2009	mild AD=17; MCI=14; HC=16	NAA, mI,	1 VOI: frontal & parietal gray-white matter	1.5T, MRSI, slice-selective IR, long TE (TR/TE/TI=1800/135/170 ms)	1) Higher mI in AD than in HC in R GM of frontal lobe, and in white matter of frontal and parietal lobe; 2) higher mI in AD than MCI in white matter of frontal lobe; 3) higher mI in MCI than HC in white matter of parietal lobe; 4) no difference in NAA.
Kantarci K	2009	MCI=151	NAA/Cr, mI/Cr Cho/Cr, NAA/mI	PCG	1.5T, PRESS, short TE (TR/TE =2000/30ms); longitudinal for an average 2.1 (0.8-6.8) years	78% MCI with hippocampal atrophy, low NAA/Cr (≤1 SD), and cortical infarctions progressed to dementia.
Pilatus U	2009	MCI=15; HC=12	tNAA (NAA+N-acetylaspartylglutamate), mI, Cr, Cho, Glx	2 VOIs: parietal WM (PWM), mid-parietal GM (PGM)	1.5T, PRESS, short TE (TR/TE =3000/22ms); longitudinal, average period of 3.4 years	1) Lower NAA (NAA/Cr) in MCI than HC at both baseline and follow-up; 2) metabolite changes in MCI-converter over time: lower tNAA and tNAA/Cr in PWM; lower tNAA and Cr in PGM; 3) baseline tNAA (or change in tNAA) positively corelated with baseline MMSE (or change of MMSE) based on all the subjects (r>0.52, p<0.05).
Jessen F	2009	dementia=130 (mild AD=98, non-AD=32); MCI=136 (AD type=70, non-AD type =66); HC=45	NAA, Cho, Cr, mI; mI/NAA, NAA/Cr	left medial temporal lobe (MTL)	1.5T, PRESS, short and long TE (TR=2000 ms, TE=272/30 ms); four centre sites	1) Lower NAA and NAA/Cr in AD than HC; 2) lower NAA in AD than in MCI of AD type and in non-AD dementia; 3) lower Cho, Cr in AD than non-AD dementia.
Parlayan E	2009	AD=20: donepezil treatment=10, rivastigmine treatment=10	NAA, NAA/Cho	not mentioned	treatment, 12 weeks, donepezil (10mg/d), rivastigmine (12mg/d)	1) Increased NAA/Cho with both treatments, compared to pre-treatment; 2) relatively more increasing effect of rivastimine on NAA/Cho than donepezil; 3) similar post-treatment improvement in MMSE with both treatments.
Wang T	2009	AD=24 (mod -severe); VD=8; HC=11	NAA, mI; NAA/Cr, mI/Cr	PCG	1.5T, PRESS, short TE (TR/TE=1500/35 ms)	1) Lower NAA/Cr and higher mI in AD than in HC; 2) lower NAA/Cr in VD than in HC; 3) NAA/Cr related with cognitive tests (p<0.05); 3) positive predictive value was 73% and negative predicitve value was 71% for identify AD from HC using NAA/Cr<1.31.
Modrego PJ	2010	AD=63 (mild-moderate): donepezi treatment=32; memantine treatment =31	NAA, Cho, Cr, mI; NAA/Cr, mI/Cr, Cho/Cr	6 VOIs: L/R temporal; L/R prefrontal; PCG; LOC	1.5T, PRESS, short TE (TR/TE=2000/35 ms); donepezil (5 mg/day for 4 wks, then 10 mg/day for 20 wks), memantine (20 mg/day for 24 wks)	1) Increased PCG NAA/Cr and Cho/Cr, increased LOC and R prefrontal mI/Cr, decreased L prefrontal NAA/Cr after donepezil treatment; 2) no significant metabolite changes in memantine group; 3) no differences in clinical scales or metabolite levels between donepezil memantine groups; more patients worsened than improved in ADAS-cog score in both group; 4) increased NAA/Cr related with improved ADAS-cog in PCG (r=-0.36, p=0.004).
Chao LL	2010	preMCI=17 (cognitive complains, not MCI; 9 for MRS); MCI=13; HC=18 (9 for MRS)	NAA/Cr, mI/Cr, NAA/mI	PCG	1.5T, STEAM, short TE (TR/TE/TM=1800/25/10 ms); one-point study	1) Lower entorhinal cortex, fusiform, and frontal gray matter volume in preMCI or MCI than in HC; 2) lower parahippocampal volume and lower PCG NAA/mI in MCI than in HC; 3) no significant differences between MCI and preMCI on any of MRI and MRS measurements; 4) significant changes in cognitive or executive tests in MCI, compared to preMCI and HC.
Li X	2010	MCI=34; HC=34	NAA, Cr; NAA/Cr	3 VOIs: prefrontal cortex (L), temporal cortex (L), parietal cortex (R)	1.5T, automated hybrid 2D CSI, short TE (TR/TE =1500/30 ms)	1) Lower NAA (NAA/Cr) in MCI than HC in L prefrontal and L temporal cortex; 2) in both L prefrontal and L temporal cortex: NAA/Cr negatively related with auditory event-related potentials in (r=-0.71~-0.53, p<0.01), and positively related with memory test scores (r=0.48~0.68, p<0.05).
Fayed N	2010	AD=33; MCI=54	NAA, Cho, Cr, mI; NAA/Cr, Cho/Cr, NAA/mI	2 voxels: PCG, occipital cortex (L)	1.5T, PRESS, short TE (TR/TE =1500/30 ms)	1) Correlation between NAA/mI and NAA/Cr with CDR and GDS in both LOC and PCG (r2=0.093-0.538, p<0.004); 2) relatively weak correlation between Cho/Cr and GDS in LOC (r2=0.039, p=0.032).
Watanabe T	2010	mild AD=70; aMCI=47; HC=52	NAA, mI, Cr, Cho	8 VOIs: L/R Hipp; anterior & posterior PDWM; PCG; occipital lobe	1.5T, PRESS, short TE (TR/TE =2000/30 ms)	1) Lower bilateral Hipp NAA in AD or aMCI than HC, and in AD than aMCI; lower bilateral posterior PDWM NAA in AD or aMCI than HC; 2) highermIin AD than HC in right Hipp; 3) lower Cho in AD than HC in bilateral Hipp, right anteiror PDWM, and bilateral posterior PDWM; in aMCI than HC in left posterior PDWM; in AD than aMCI in left Hipp; 4) lower Cr in AD than HC in bilateral Hipp; in aMCI than HC in left Hipp and left posterior PDWM.
Schott JM	2010	moderate AD =42, HC=22 at baseline	NAA/Cr, Cho/Cr, mI/Cr, NAA/mI	PCG	1.5T, PRESS, short TE (TR/TE =2000/30 ms); longitudinal, six times of MRS scans over 24 month, 71% patents enrolled took ACEI	1) Lower NAA/Cr & NAA/mI, higher mI/Cr in AD than HC at baseline; 2) baseline NAA/mI distinguish AD from HC: 83% sensitivity, 77% specificity; 3) NAA/Cr and NAA/mI in AD decreased over time, no metabolite change in HC over time; 4) NAA/mI declined faster in AD than in HC (p=0.014); 5) Between-subject standard deviation for NAA/mI was 0% for HC and 3.5%/year for AD; within-subject standard deviation for one year, two-time-point study was 9.2%/year for both AD and HC.
Westman E	2010	mild AD=30; HC=36	NAA/Cr, Cho/Cr, mI/Cr	Hippocampus	1.5T, PRESS, short TE (TR/TE =1500/35 ms)	Combining MRS and MRI improved AD/HC identification: sensitivity=97%, specificity=94%, compared with using MRI or MRS alone: sensitivity=93%, 76%, specificity =86%, 83%.
Didic M	2010	aMCI=28 (16 with and 12 without impaired visual recognition) HC=28	NAA/mI	12 VOIs in the MTL region	1.5T, MRSI, short TE inversion recovery 2D spin echo sequence (TI/TE/TR=150/22/1500 ms); one-point MRS, 6-year clinical follow-up aMCI	1) MCI vs HC: lower NAA/mI in right MTL; 2) aMCI with impaired visual recognition vs HC: lower NAA/mI in left anterior MTL; 3) the level of NAA/mI in anterior MTL corelated wtih visual memory performance (r=0.309-0.351, p<0.05) in the sample; 4) visual memory performance predicted 6-year AD-conversion (81.8% sensitivity and specificity).
Sailasuta N	2011	mild AD=2; MCI=2; HC=2	NAA/Cr, mI/Cr, NAA/mI; bicarbonate production	2 VOIs: PCG white matter, PCG gray matter	1.5T, 1H MRS and 13C MRS, short TE; TR/TE =1500/35ms for 1H MRS)	1) Reduced NAA/mI related with change of cognitive test (p=0.01); 2) bicarbonate production rate related with cognitive test score (r2=0.91) and mI/Cr (r2=0.75).
Fayed N	2011	AD=30 (mild -moderate); MCI=68; HC=26	NAA, Glu	PCG	1.5T, PRESS, short TE (TR/TE =2000/35ms); donepezil (10AD, 10 mg/day), memantine (10 AD, 20 mg/day), 24 weeks	1) Lower NAA in AD or MCI than HC; 2) lower Glu in AD than MCI or HC.3) Increased significant Glx/Cr (p=0.007) and decreased NAA (p=0.04) after treatment regardless drug type.
Zimny A	2011	AD=30 (moderate); aMCI=23; HC=15	NAA/Cr, Cho/Cr, mI/Cr, mI/NAA, mI/Cho	PCG	1.5T MRS, PRESS, short TE (TR/TE=1500/35 ms); PWI; DTI	Diagnosis accuaracy of AD and aMCI vs HC using MRS were 0.82 and 0.47 respectively, no better than using DTI or PWI.
Walecki J	2011	MCI=31; (stable SD=8, progression DP =13, AD=10)	Ratios of NAA, Cho, mI, Glx /Cr and /H2O,	6 VOIs: L/R frontal, temporal	1.5T, PRESS, short TE (TR/TE =1500/35 ms); clinical follow-up MCI	1) SD vs DP: lower NAA/H2O & mI/H2O in L temporal & L frontal in SD; 2) AD vs DP: lower NAA/Cr in L frontal in AD; 3) AD vs SD: L temporal lower mI/Cr, R temporal lower Cho/Cr, R temporal medial lower Glx/H2O in AD.
Watanabe T	2012	AD=67; aMCI=42; HC=54	NAA and mI concentration	Hippocampus (L/R); PCG	1.5T, PRESS, (TR/TE=2000/30ms); linked to cognition	Strong association of Hipp (especially L) NAA and mI with memory dysfunction in aMCI and AD; less significant for PCG NAA and mI.

**Table 2.** *In vivo* single voxel proton magnetic resonance spectroscopy studies on Alzheimer’s disease at high field (≥3.0 T).

First Author	Year	Subjects	Metabolites	Locations	Methods	Main Findings
Hattori N	2002	AD=9 (moderate); HC=12	NAA/Cr, Cho/Cr, mI/Cr, Glx/Cr	2 VOIs: PCG, left parieto-occipital white matter	3T, PRESS, short TE (TR/TE=6000/25ms)	1) AD vs. HC, lower NAA/Cr in both voxels. 2) lower Glx/Cr in PCG only. 3) levels of NAA/Cr and Glx/Cr in PCG were correlated in AD (r=0.722, p<0.05).
Kantarci K	2003	mild AD=20; MCI=20; HC=41	NAA/Cr; Cho/Cr; mI/Cr; Glu/Cr; (Gln+Glu)/Cr; NAA/mI	PCG	3T and 1.5T, PRESS, short or long TE (TR=2000 ms, TE=135ms for NAA, Cho, Cr, TE=30ms for other metabolites)	1) Improved metabolite ratios at 3T than at 1.5T; 2) Glx at 3T; 3) higher Cho/Cr and mI/Cr, lower NAA/Cr and NAA/mI in MCI vs HC only at 1.5T; same metabolite differences in AD vs HC at both 3T and 1.5T; 4) accuracy of 0.81 for 1.5T, 0.70 for 3.0T in differentiate AD from HC using NAA/mI.
Kaiser LG	2005	younger HC=10; older HC=14	NAA, mI, sI, Tau; NAA/Cr, mI/Cr, sI/Cr, Tau/Cr	corona radiata white matter	4T, STEAM, short TE (TR/TE/TM= 2000/15/10ms)	1) Elevated mI (mI/Cr), sI (sI/Cr) and lowered NAA (NAA/Cr) in older than in younger subjects; 2) levels of mI and sI were correlated (r=0.4, p=0.06). 3) Comparatively lower relaibility using sI (than NAA).
Mihara M	2006	AD=8 (moderate); FTD/Pick=10 HC=14	NAA/Cr, Cho/Cr, mI/Cr	4 VOIs: PCG, ACG, parieto-occipital white matter (POWM), prefrontal white matter (PFWM)	3T, PRESS, short TE (TR/TE=6000/25ms)	1) AD vs HC: lower NAA/Cr in PCG; 2) FTD/Pick vs HC: lower NAA/Cr in PCG, ACG, POWM; higher mI/Cr in PCG and POWM; lower Cho/Cr in PWM; 3) AD: lower posterior/anterior NAA ratio.
Griffith HR	2007	mild AD=15; aMCI=26; HC=19	sI/Cr , NAA/Cr, Cho/Cr, mI/Cr,	PCG	3T, PRESS, short TE (TR/TE=2000/32ms); ACEI treatment in 12 AD and 13 aMCI	1) Higher sI/Cr and mI/Cr, lower NAA/Cr in AD than in HC; 2) higher mI/Cr in AD than MCI, 3) higher mI/Cr in MCI than in HC; 4) sI/Cr correlated with NAA/Cr (r=-0.3) and with mI/Cr (r=0.24); 5) no correlations with age; 6) no significant effect of ChEI; 7) sI/Cr was related with executive functioning in AD (r=-0.6, p<0.03).
Griffith HR	2007	MCI=26; HC=20	NAA/Cr, Cho/Cr, mI/Cr	PCG	3T, PRESS, short TE (TR/TE=2000/32 ms)	mI/Cr in PCG was negatively correlated with executive function (r2=0.26, p=0.005).
Bartha R	2008	mild AD=10; HC=5	NAA, Glu,Cho, Cr, mI; NAA/Cr, NAA/Cho, mI/Cr, mI/Cho, Cho/Cr, Glu/NAA	right hippo-campus	4T, LASER, TR/TE=3200/46ms; donepezil treatment 16 weeks; one year follow-up for HC	1) Decreased NAA, Cho, NAA/Cr, Cho/Cr, and mI/Cr in AD after treatment; 2) increased mI/Cho in HC over one year.
Caserta MT	2008	mild memory impairment (MMI)=8; mild AD=6; HC=17	NAA/Cr; mI/Cr; Cho/Cr	3 VOIs: PCG; right and left hippo-campus	3T, PRESS, short TE (TR/TE=2000/30ms)	1) Reduced NAA/Cr in MMI vs. HC in right Hipp; 2) reduced NAA/Cr in AD vs, HC in bilateral hippocampus.
Griffith HR	2008	mild AD=22; Parkinson's disease (PD)=12; HC=61	NAA/Cr; mI/Cr; Cho/Cr, Glu/Cr	PCG	3T, PRESS, short TE's (TR=2000 ms, TE=32ms for NAA, Cho, mI; TE=80 ms for Glu)	1) lower NAA/Cr and higher Cho/Cr and mI/Cr in AD than in HC; 2) lower NAA/Cr and Glu/Cr in PD than in HC; 3) lower Glu/Cr in PD than in AD; 4) NAA/Cr and Glu/Cr positively corelated with while mI/Cr and Cho/Cr negatively corelated with cognitive evaluation (p<0.05).
Glodzik L	2008	AD=3; MCI=4; HC=9 (3 treated)	NAA/Cr, Glu/Cr	hippo-campus	3T, MRSI (0.5cm3, VOI=7x9x2cm3); PRESS (water suppression through T1 effects, short TE: TR/TE=1600/39 ms; memantine treatment (5mg/day to 10mg/day 4+ weeks; 20mg/day 20 weeks)	1) Glu/Cr decrease was slower in treat-group than in nontreat group in left Hipp; 2) increased Glu/Cr in non-treated group with time; trend lower Glu/Cr in treated group with time; 3) no group or over-time difference in NAA/Cr.
Rupsingh R	2009	mild AD=23; MCI=12; HC=15	NAA, Glu,Cho, Cr, mI; NAA/Cr, Glu/Cr, Glu/NAA, Glu/mI	right hippo-campus	4T, LASER, short TE (TE=46 ms)	1) A decrease of Glu (absolute or ratios) in AD than in HC; 2) a decrease of NAA/Cr in AD than in HC; 3) a decrease in Glu/mI in AD than in MCI; 4) no metabolite difference MCI vs HC; 5) NAA correlated with cognition, specially in MCI (r=0.64,p<0.05); 6) AD vs HC discrimination performance was 0.71 by NAA/Cr, 0.80 by Glu, 0.94 by NAA/Cr+Glu+mI.
Wang Z	2009	AD=16 (severe); MCI=16; HC=16	NAA, Cho, Cr, mI; NAA/Cr,Cho/Cr,mI/Cr,mI/NAA	PCG; hippo-campus	3T, PRESS, short TE TR/TE=2000/30ms for SV MRS (TR/TE=1700/30ms for MRSI)	1) Increased mI/NAA in AD than in HC or MCI in both Hipp and PCG; 2) increased mI/NAA in MCI than in HC in Hipp, not PCG; 3) increased mI/Cr and decreased NAA/Cr in AD or MCI than in HC in Hipp, not PCG; 4) decreased NAA/Cr in AD than in MCI in Hipp, not in PCG; 5) highest mI/NAA in AD in Hipp, then MCI in Hipp, then AD in PCG, finally MCI in PCG; 6) mI/NAA correlated with cognition in both locations (r>0.84, p<0.001).
Griffith HR	2010	MCI=29; HC=42	NAA/Cr, Cho/Cr, mI/Cr	PCG	3T, PRESS, short TE (TR/TE=2000/32 ms)	1) Higher mI/Cr and Cho/Cr in MCI than in HC; 2) no group difference in NAA/Cr; 3) NAA/Cr correlated with decision making capacity in MCI (r=0.46, p<0.05).
Penner J	2010	mild AD=10	NAA, Glu,Cho, Cr, mI; NAA/Cr, Glu/Cr, Glu/NAA, Glu/mI	right hippo-campus	4T, LASER, short TE (TR/TE=3200/46ms); galantamine treatment (8 mg/day 4 weeks; 16 mg/day 12 weeks)	1) Glu (as absolute or as ratios) increased after treatment; 2) a trend of NAA and Cr decrease, but not significant. 3) changes of Glu correlated with changes of cognition (r2>0.49, p<0.05).
Weaver KE	2010	aMCI=12	lactate	PCG	3T, 2D J-resolved MRS sequence, short TE (TE=30ms)	Lactate level was negatively correlated with cognitive performance (p<=0.05).
Ashford JW	2011	AD=13 (mild-moderate; memantine treatment=7)	NAA/Cr	3 VOIs: PCG; left cerebral cortex; inferior parietal	3T, PRESS, short TE (TR/TE=2000/35ms); memantine treatment (n=7; 54 weeks)	1) No NAA/Cr differences between treat and placebo groups at both baseline and follow-up; 2) changes in NAA/Cr correlated with changes in ADAS-cog for all the subjects; 3) baseline NAA/Cr correlated with age and verbal fluency (p<0.05).
Emir UE	2011	younger HC=22; older HC=22	ascorbate (Asc), glutathione (GSH), lactate	occipital (midline)	4T, double edited MEGA-PRESS, long TE (TR/TE= 4500/122ms)	1) Lower GSH in elderly than in young subjects; 2) no significant age-associated Asc change; 3) increased lactate in older adults.
Henigsberg N	2011	AD=12 (mild /moderate)	NAA/Cr	DLPFC	3T, long TE (272ms); donepezil treatment 10 mg/day 26 weeks	An increase in NAA/Cr was obseved in 10/12 indiivduals post treatment. Marginal pre- vs post- treatment difference at group level (p=0.043).
Kantarci K	2011	HC (older) =311	mI/Cr, Cho/Cr,	PCG	3T, 1H MRS, PRESS, short TE (TR/TE=2000/30ms); PiB-PET	1) Elevated mI/Cr and Cho/Cr associated with higher retention of Pittsburgh compound B (p=0.003, p=0.022 respectively); 2) higher Cho/Cr was associated with worse cognitive performance, independent of Aβ load.
Lim TS	2012 a	AD=23; aMCI=16; HC=22	NAA/Cr	6 VOIs: bileteral PCGs and surounding regions	3T, PRESS, long TE (TR/TE=2000/144 ms)	1) NAA/Cr correlated with verbal memory for immediate recall and delayed recall; 2) No difference in NAA/Cr was found between the VOIs on the left and right sides.
Lim TS	2012b	AD=36; aMCI=19; HC=23	NAA/Cr, mI/Cr	2 VOIs: PCG, ACG	3T, PRESS, short TE (TR/TE=2000/9.177 ms)	1) Lower PCG NAA/Cr in AD and in aMCI than in HC; 2) higher ACG mI/Cr in AD than in amnestic MCI and in HC; 3) PCG mI/Cr correlated with the MMSE; 4) ACG mI/Cr correlated with the neuropsychiatric inventory.
Mandal PK	2012	probable AD=14; MCI=11; older HC= 15; younger HC=45	GSH	left/right frontal lobe VOIs in MCI & AD (more for HC)	3T, MEGA-PRESS (TR/TE= 2500/120ms, 4.40ppm 180° pulse refocusing)	1) Lower mean GSH levels in AD than in younger HC in the frontal lobe VOIs for both sexes. 2) a trend of GSH reduction from MCI to AD. 3) in younger subjects, some gender, spatial, and lateral differences in the level of GSH.
Seo SW	2012	aMCI=13; HC=11	NAA/Cr, Cho/Cr	4 VOIs: PCG; Hipp; left entorhinal occipital WM;	3T, PRESS, short TE (TR/TE=2000/40ms); 3-yr clinical follow-up for MCI-AD	1) Lower NAA/Cr in amnestic MCI than HC in the entorhinal cortex, especially in MCI-AD converters; 2) no significant group difference in other regions.
Wang T	2012	probable AD=47; aMCI=32; HC=56	NAA/Cr,Cho/Cr,NAA/mI	3 VOIs: PCG; left and right hippo-campus	3T, PRESS, short TE (TR/TE=1500/35ms)	1) AD vs. HC: NAA/mI, NAA/Cr, mI/Cr, Cho/Cr all differed in PCG; NAA/mI and mI/Cr differed in left hipp; only NAA/mI differed in right hipp; 2) AD vs. MCI: all the ratios still differed, but only in PCG, not in hipp; 3) MCI vs. HC: NAA/mI, NAA/Cr differed in PCG; mI/Cr in left hipp; 4) aMCI / HC discrimination by PCG NAA/Cr ≤ 1.50: sensitivity=93.8%, specificity=92.9%, by NAA/mI≤2.72: sensitivity=75.0%, specificity=80.0%.
Gordon ML	2012	AD=11 (mild -moderate); HC=28	NAA, NAA/Cr, NAA/Cho, mI, NAA/mI	precuneus-PCG	3T, PRESS, short TE (TR/TE=1600/30 ms); 24-week ACEI treatment, followed by 24-week memantine treatment	1) Higher mI/Cr and lower baseline NAA, NAA/Cr , NAA/Cho, and NAA/mI in AD than in HC at baseline; 2) baseline NAA/Cr, mI/Cr, and NAA/mI correlated with cognitive/functional testing scores; 3) when memantine was added to a ACEI, there was an increase in mI and a decrease in NAA/mI, but no other change in metabolites or in neurocognitive measures.
Bittner DM	2013	AD=19 (mild /moderate); older HC=17	NAA/Cr, Cho/Cr, mI/Cr	3 VOIs: right Hipp; parietal PCG;	3T, PRESS (TR/TE= 2500/135ms); linked to CSF A*β*42 and pTau	1) Decreased NAA/Cr in all VOIs in AD, especially in Hipp; 2) highest AD/HC classification using Hipp NAA/Cr (over .94/.92 for sen/spe); 3) NAA/Cr in Hipp and parietal VOIs and mI/Cr in PCG were associated with CSF biomarkers and with cognition.
Friedman SD	2013	MCI=17; adult HC=13 (55-87 yrs)	Glu/Cr, GABA/Cr, mI/Cr, NAAG/NAA,	3 VOIs: PCG, dorsolateral frontal and posterior parietal lobes	3T, PRESS, TR/TE=2000/35~380ms; Randomized, double-blind, placebo-controlled substudy; Growth hormone-releasing hormone (GHRH) administration (20 weeks)	1) GABA increased in all the three VOIs, NAAG increased in dorsolateral frontal lobe, mI decreased in PCG, no Glu changes, similar in MCI and normal aging; 2) treatment-related chnges in GABA in PCG positively correlated with serum insulin-like growth factor 1, and tended to be negatively correlated with mI; 3) a favorable treatment effect on cognition was observed (but not significantly related with neurochemical changes).

## ABBREVIATIONS

ACG: = Anterior Cingulated Gyrus
AD: = Alzheimer’s Disease
aMCI: = Amnestic MCI
A*β*: = Amyloid beta protein
CDR: = Clinical Dementia Rating
ChEI: = Cholinesterase Inhibitors
Cho: = Choline
CIND: = Cognitive Impairment No Dementia
Cr: = Creatine
CSF: = Cerebrospinal Fluid
DLPFC: = Dorsolateral Prefrontal Cortex
DTI: = Diffusion Tensor Imaging
FDG-PET: = Fludeoxyglucose (18F) - Positron Emission Tomography
FTD-Pick: = Frontotemporal Dementia-Pick complex
GABA: = Gama (γ)-Aminobutyric Acid
GDS: = Geriatric Depression Scale
Glc: = Glucose
Gln: = Glutamine
Glu: = Glutamate
GM: = Grey Matter
GSH: = Glutathione
HC: = Healthy Control
Hipp: = Hippocampus
Lac: = Lactate
LASER: = Localization by Adiabatic Selective Refocusing
LBD: = Lewy Body Dementia
MCI: = Mild Cognitive Impairment
*m*I: = myo-Inositol
MRI: = Magnetic resonance imaging
MRS: = Magnetic Resonance Spectroscopy
MRSI: = Magnetic Resonance Spectroscopy Imaging
MTL: = Medial Temporal Lobe
NAA: = N-Acetyl Aspartate
NAAG: = N-Acetyl Aspartyl-Glutamate
NFT: = Neurofibrillary Tangles
PCG: = Posterior Cingulate Gyrus
PD: = Parkinson's Disease
PRESS: = Point Resolved Spectroscopy Sequence
RF: = Radio Frequency
*s*I: = scyllo-Inositol
SNR: = Signal to Noise Ratio
STEAM: = Stimulated Echo Acquisition Mode Sequence
SV ^1^H MRS: = Single Voxel Proton Magnetic Resonance Spectroscopy
T: = Tesla
Tau: = Taurine
TE: = Echo Time
TR: = Repetition Time
VOI: = Voxel of Interest
WM: = White Matter
